# *Daphnia* predation on the amphibian chytrid fungus and its impacts on disease risk in tadpoles

**DOI:** 10.1002/ece3.777

**Published:** 2013-09-23

**Authors:** Catherine L Searle, Joseph R Mendelson, Linda E Green, Meghan A Duffy

**Affiliations:** 1 School of Biology, Georgia Institute of TechnologyAtlanta, Georgia, 30332; 2 Department of Ecology and Evolutionary BiologyUniversity of Michigan, 2019 Kraus Natural Science Building, 830 North University, Ann Arbor, MI 48109-1048; 3 Zoo Atlanta800 Cherokee Ave. SE, Atlanta, Georgia, 30315

**Keywords:** *Batrachochytrium dendrobatidis*, chytridiomycosis, eutrophication, *Lithobates sphenocephalus*, parasites, trophic interactions

## Abstract

Direct predation upon parasites has the potential to reduce infection in host populations. For example, the fungal parasite of amphibians, *Batrachochytrium dendrobatidis* (Bd), is commonly transmitted through a free-swimming zoospore stage that may be vulnerable to predation. Potential predators of Bd include freshwater zooplankton that graze on organisms in the water column. We tested the ability of two species of freshwater crustacean (*Daphnia magna* and *D. dentifera*) to consume Bd and to reduce Bd density in water and infection in tadpoles. In a series of laboratory experiments, we allowed *Daphnia* to graze in water containing Bd while manipulating *Daphnia* densities, *Daphnia* species identity, grazing periods and concentrations of suspended algae (*Ankistrodesmus falcatus*). We then exposed tadpoles to the grazed water. We found that high densities of *D. magna* reduced the amount of Bd detected in water, leading to a reduction in the proportion of tadpoles that became infected. *Daphnia dentifera*, a smaller species of *Daphnia*, also reduced Bd in water samples, but did not have an effect on tadpole infection. We also found that algae affected Bd in complex ways. When *Daphnia* were absent, less Bd was detected in water and tadpole samples when concentrations of algae were higher, indicating a direct negative effect of algae on Bd. When *Daphnia* were present, however, the amount of Bd detected in water samples showed the opposite trend, with less Bd when densities of algae were lower. Our results indicate that *Daphnia* can reduce Bd levels in water and infection in tadpoles, but these effects vary with species, algal concentration, and *Daphnia* density. Therefore, the ability of predators to consume parasites and reduce infection is likely to vary depending on ecological context.

## Introduction

Host–parasite interactions, while often studied in isolation, are affected by a multitude of direct and indirect effects from other members of the community (Omacini et al. 2001; Lafferty 2004; Lafferty et al. 2006). Predators are one notable community member that can strongly impact infectious diseases. In some cases, predators can increase infection in their prey (e.g., through impacts on host immune function or host traits; Ramirez and Snyder 2009; Duffy et al. 2011). In other cases, predators can decrease disease risk in their prey (e.g., by decreasing prey population sizes or directly consuming infected hosts; Packer et al. 2003; Keesing et al. 2006; Duffy et al. 2005). Predators can also impact disease risk in non-prey species via consumption of disease vectors or free-living stages of parasites (Grutter 1996; Nelson and Jackson 2006; Orlofske et al. 2012). Therefore, predators have the potential to alter rates of infection in both prey and nonprey species through a variety of routes.

The potential role of predation in reducing infectious disease risk is of particular interest when applied to medicine and conservation. Indeed, manipulation of predator densities has been suggested as a potential conservation measure (Packer et al. 2003). In some cases, the focus is on the potential for predators to reduce density of vectors. For example, augmentation of populations of mosquito predators has been suggested as a way to control mosquito-borne parasites such as malaria (Nelson and Jackson 2006; Howard et al. 2007). In other cases, the focus has been on the potential for predators to directly prey upon the parasites. It is this latter scenario that is the focus of the experiments reported here. We studied the potential for predation upon the fungus *Batrachochytrium dendrobatidis* (Bd), which has caused population declines and extirpations of amphibians around the globe (Bosch et al. 2001; Lips et al. 2006; Skerratt et al. 2007; Wake and Vredenburg 2008). Predation by zooplankton on free-swimming Bd zoospores has been suggested as a possible method for biocontrol of this fungus (Buck et al. 2011). Our study further evaluates this possibility.

There are reasons to expect that free-swimming Bd might be vulnerable to predation. First, Bd is generally transmitted through an aquatic zoospore stage that swims through water to infect new hosts (Longcore et al. 1999). The length of time that zoospores can remain infectious is context-dependent; Piotrowski et al. (2004) found that 95% of zoospores stop moving after just 24 h in distilled water, while Johnson and Speare (2003) reported motile zoospores in lake water after 7 weeks. Given the potential for a long free-swimming stage, Bd zoospores may be at risk of direct predation during this infectious period. Second, many bodies of water contain numerous microcrustaceans that have the potential to consume Bd zoospores. For example, *Daphnia* are generalist grazers of algae, bacteria, cyanobacteria, protozoans, fungi, and detritus. One species of *Daphnia* (*D. galeata hyalina*) has been shown to consume zoospores of a pathogenic chytrid of diatoms, reducing infection in the hosts (Kagami et al. 2004). Bd zoospores are generally 3−5 μm in diameter (Longcore et al. 1999), which is within the preferred range of food size for many *Daphnia* (Burns 1968; Geller and Muller 1981). Therefore, *Daphnia* are good candidates for predators of Bd.

Three previous studies have directly investigated the potential for *Daphnia* to impact Bd. In a laboratory experiment, Buck et al. (2011) demonstrated that *Daphnia* can consume Bd zoospores. However, the ability of *Daphnia* to digest those zoospores was not tested, and previous studies have shown that passage through a *Daphnia* gut can actually increase growth of some organisms (Porter 1976). Therefore, it is possible that Bd zoospores can be ingested by *Daphnia* but not digested. Two additional laboratory studies demonstrated that *Daphnia* reduce the number of zoospores detected in water samples (Woodhams et al. 2011; Hamilton et al. 2012), but a mesocosm experiment did not find changes in infection rates in tadpoles (Hamilton et al. 2012). Each of these studies only investigated one species of *Daphnia*, but *Daphnia* species vary in body size, which can influence feeding preferences and rates (Burns 1968; Hall et al. 2007). Additionally, the laboratory studies testing for *Daphnia* predation upon Bd (Woodhams et al. 2011; Hamilton et al. 2012) combined *Daphnia* and Bd zoospores in clean water without the presence of other food sources for *Daphnia*. Other studies have demonstrated that gut passage time and food assimilation efficiency in *Daphnia* change with food concentration (DeMott et al. 2010). Therefore, the presence of alternative food resources (as is the case in natural communities containing Bd and *Daphnia*) may alter the consumption and digestion rates of *Daphnia* on Bd zoospores.

In this study, we tested the ability of *Daphnia* to consume Bd zoospores and reduce both environmental levels of Bd and infection in tadpoles. In a series of laboratory experiments, we varied density of two species of *Daphnia* (*D. magna* and *D. dentifera*) to compare the effectiveness of each species at consuming Bd. Additionally, as algal levels can vary greatly between water bodies, we manipulated the density of suspended algae (food for *Daphnia*) to determine its effects on Bd consumption by *D. dentifera*. Our goal was to understand the impact of zooplankton predation on Bd levels in the environment and hosts.

## Methods

### Study organisms

To ensure that amphibians used in the experiment were not previously infected with Bd, we collected southern leopard frogs (*Lithobates sphenocephalus*; formerly *Rana sphenocephala*) as eggs from Fall Line Sandhills Wildlife Management Area near Butler, Georgia, USA. We collected partial clutches of 11 egg masses and immediately brought them to the laboratory to be reared in 37.8 L aquaria filled with aged tap water treated with tap water conditioner (API). The laboratory was maintained at ∼20°C with a 12:12 light:dark photoperiod.

**Figure 1:**
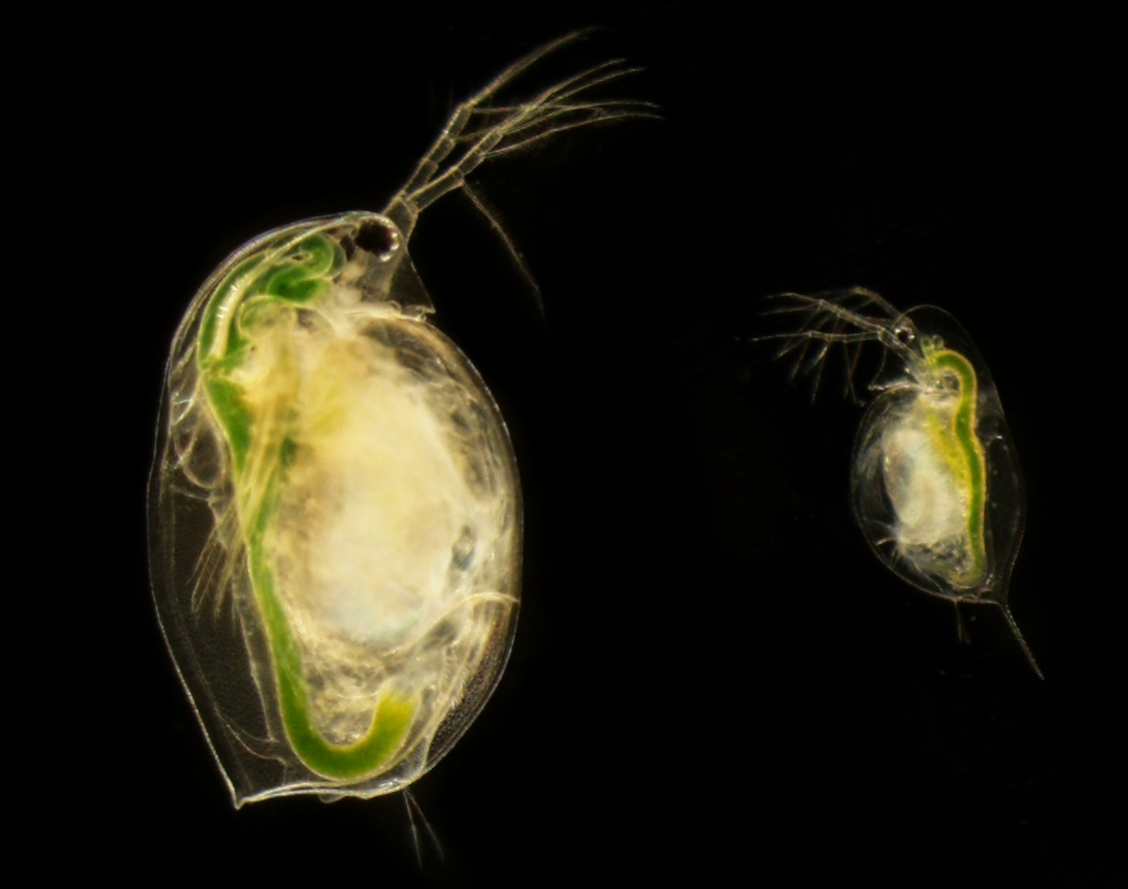
Photograph of *Daphnia* species used in this experiment. The individual on the left is a *D. magna* adult female, and the individual on the right is a *D. dentifera* adult female.

We used *D. magna* isolated from Kaimes Pond in Scotland, UK, and *D. dentifera* from Midland Lake in Indiana, USA. We chose these species because they vary in body size; *D. magna* is a large Eurasian species and can grow up to ∼5 mm in length (Bottrell et al. 1976); *D. dentifera* is a smaller North American species that grows to ∼2 mm (Hall et al. 2007; Fig. 1). We used one isofemale line for each species to reduce variation among individual *Daphnia*. *Daphnia* were 11−14 days old at the beginning of each experiment. We used Bd Strain SR-810, which was originally isolated from a *Lithobates catesbeianus* tadpole from South Carolina (Schloegel et al. 2009). For use in the experiment, we cultured Bd onto 1% tryptone agar petri dishes and allowed them to grow for 4−7 days.

This study was conducted in accordance with the recommendations in the Guide for the Care and Use of Laboratory Animals of the National Institutes of Health. The Institutional Animal Care and Use Committee (IACUC) at Georgia Institute of Technology was informed of this study, but did not require animal care protocols for the early stage tadpoles used in these experiments.

### Experimental design

We performed three experiments manipulating *D. magna* density, *D. dentifera* density, grazing period, and algal density. Each experiment had a similar protocol. For all experiments, we placed *Daphnia* into 400 mL glass beakers filled with 250 mL of media (50% filtered lake water and 50% artificial *Daphnia* medium; Kluttgen et al. 1994). We then flooded Bd-inoculated petri dishes with 15 mL media for 30 min. This method allows for zoospores to release into the water without dislodging sporangia that are attached to the agar (Boyle et al. 2004; Searle et al. 2011). We pooled inoculum from these dishes and quantified zoospore densities using a hemocytometer. We then added ∼2.0 × 10^4^ Bd zoospores to each beaker to create a concentration of 80 zoospores per mL in the beakers. Beakers were only inoculated with Bd once, at the beginning of the experiment. We fed the green alga *Ankistrodesmus falcatus* to *Daphnia* immediately after addition of Bd zoospores at densities described below. Additionally, we established three beakers that were treated in the same manner as the others (with 0, 1, and 5 *Daphnia* per beaker) except Bd inoculum was not added to the water. The samples from unexposed beakers served as negative experimental controls for infection analysis (described below). *Daphnia* grazed for 5, 24, or 72 h (see below). We chose these times because previous studies have demonstrated that *Daphnia* can reduce Bd densities in water after 5 or 72 h (5 h: Hamilton et al. 2012; 72 h: Woodhams et al. 2011). We fed *A. falcatus* to *Daphnia* in the 5- or 24- h grazing period once immediately after addition of Bd zoospores, and we fed *A. falcatus* to *Daphnia* in the 72-h treatments three times; immediately after addition of Bd zoospores, then every 24 h. Under the conditions in this experiment, individual *D. dentifera* can filter over 10 mL of water per day (Hall et al. 2010), while *D. magna* can filter even greater amounts of water due to their larger body size. Thus, in the treatments with 25 *Daphnia* per beaker, it is highly likely that the entire contents of a beaker would have filtered at least once during a 24-h period.

After the grazing period, we removed all *Daphnia* (including any offspring born during the experiment) from the beakers using a glass Pasteur pipette. For the 72-h grazing period, we also removed offspring every 24 h to reduce variation among replicates. After removal of all *Daphnia*, we stirred each beaker vigorously using a glass stir rod and removed 1.5 mL water from 3.5 cm below the water's surface and placed it into a microcentrifuge tube. After taking water samples, we added one *L. sphenocephalus* tadpole to each beaker where they remained for 24 h. We then euthanized each tadpole in an individual container containing an overdose of buffered MS-222. The MS-222 solution also acted as a wash to remove any zoospores that may have been in the tadpole's water or on the surface of the tadpole, but not infecting them. By euthanizing tadpoles immediately after the 24-h exposure, we were able to focus on the effects of *Daphnia* on Bd transmission (rather than the progression of infection); additional rounds of infection were unlikely to develop in this time period, because the life cycle of Bd takes 5 days under optimal conditions (Johnson and Speare 2003). We preserved tadpoles individually in 95% ethanol. Tadpole mass among experiments is compared in Figure S2 and Data S1.

Experiments 1 and 2 manipulated the density of *D. magna* and *D. dentifera* with two grazing periods, 5 h or 72 h (3 days). For experiment 1, we used six density treatments with 0, 1, 5, 10, or 25 *D. magna* per beaker and one treatment with 25 *D. dentifera* per beaker. This is equivalent to 0, 4, 20, 40, and 100 *D. magna* per L and 100 *D. dentifera* per L. Each treatment was replicated 10 times. Experiment 2 had the same design as experiment 1, but focusing only on *D. dentifera* at densities of 0, 1, 5, 10, and 25 *D. dentifera* per beaker (0, 4, 20, 40, and 100 *D. dentifera* per L). For experiments 1 and 2, we added 2.5 × 10^6^ cells of *A. falcatus* to each beaker to create a concentration of 1 × 10^4^ cells/mL.

For experiment 3, we manipulated the density of *Daphnia* and the density of suspended algae. We used two densities of *D. dentifera* (0 or 25 individuals per beaker; 0 or 100 *D. dentifera* per L) and two densities of *A. falcatus* (1 × 10^4^ cells/mL [“high food”] and 80 cells/mL [“low food”]). The density of *A. falcatus* in the high-food treatment was the same as in experiments 1 and 2, while the low-food treatment had a density of algal cells equal to the concentration of Bd zoospores in the water. We allowed *Daphnia* to graze for 24 h in experiment 3, as an intermediate time period of the grazing times from experiments 1 and 2.

### Zoospore quantification

To quantify Bd concentrations in water and tadpoles, we performed quantitative PCR (qPCR) on water samples and tadpole mouthparts. Extractions on water samples followed Hamilton et al. (2012) with modifications. Briefly, we centrifuged water samples for 10 min at 16k and removed all but 50 μL supernatant. We then added 150 μL PrepMan Ultra (Applied Biosystems, Foster City, CA) and 40 mg silica/zirconium beads (Biospec Products, Bartlesvill, OK) and homogenized tubes for 45 s on a Vortex-Genie 2 vortex (MO BIO Laboratories Inc., Carlsbad, CA) then centrifuged at 13k for 30 s. We repeated homogenizing and centrifugation then heated samples to 100°C for 10 min. After cooling for 5 min, we centrifuged samples for 3 min at 13k, collected supernatant, and diluted it to a 10% solution with nuclease-free water. We extracted tadpole samples according to Boyle et al. (2004) except using 60 μL Prepman Ultra instead of 40 μL. We performed qPCR according to Boyle et al. (2004) on a Mastercycler ep realplex (Eppendorf) and analyzed each sample in triplicate. We included a no-template control (nanopure water instead of amphibian sample) in each qPCR plate and never observed amplification in these controls. If a sample tested positive for Bd in only one replicate, we reanalyzed the sample. We considered a sample positive for Bd if we detected Bd in 2 of 3 replicates (run once) or 3 of 6 replicates (run twice). We performed qPCR on all water and tadpole samples from each experiment including the three samples from each experiment that were not exposed to Bd. All of the unexposed samples were negative for the presence of Bd.

### Statistical analyses

We performed all statistical analyses in R version 2.15.1 (R Core Development Team 2012). Our infection data were in the form of genome equivalents per sample and contained a large number of zeros. We therefore fit zero-inflated negative binomial models to our infection data using the “pscl” package. This allowed us to model both the presence of Bd in a sample and the amount of Bd detected (Zuur et al. 2009). For each experiment, we fit a separate model for water and tadpole samples. For experiments 1 and 2, our initial models included *D. magna* or *D. dentifera* density (respectively), grazing period (5 vs. 72 h), and the interaction between these two predictors. For experiment 3, our initial models included food level, *Daphnia* density, and the interaction term. We then dropped predictors from the models based on likelihood ratio tests (package “lmtest” Zuur et al. 2009).

To compare the effects of *D. magna* versus *D. dentifera* density in experiment 1, we performed generalized linear models (GLMs) on the proportion of samples positive for Bd (binomial GLM with a logit link) in three treatments: 0 *Daphnia*, 25 *D. magna*, and 25 *D. dentifera*. For significant effects, we then performed GLMs comparing two treatments at a time and corrected for multiple comparisons with a Bonferroni correction. For the samples that were positive for infection, we also performed an ANOVA on the log amount of Bd detected for water and tadpole samples in each experiment using the same predictors. When ANOVA revealed a significant effect, we followed with a Tukey's HSD test to directly compare treatments.

## Results

### Experiment 1

**Figure 2:**
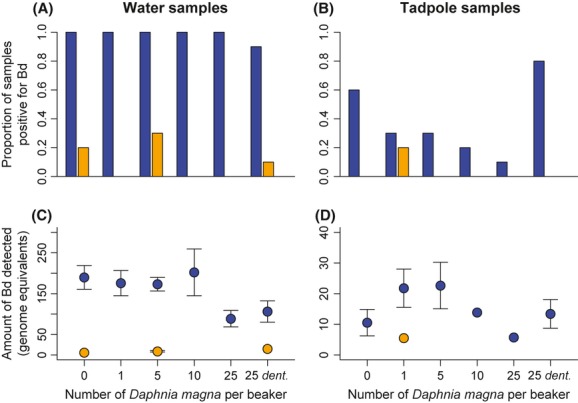
Results from experiment 1. Infection prevalence and amount of *Batrachochytrium dendrobatidis* (Bd) detected in samples from experiment 1. Numbers on the *x*-axis indicated the number of *Daphnia magna* per beaker while “25 *dent*.” indicates the treatment with 25 *D. dentifera* per beaker. Dark (blue) bars and points represent the 5-h grazing period and light (orange) bars and points represent the 72-h grazing period. After the grazing period, we exposed all tadpoles to grazed water for 24 h. The proportion of samples positive for Bd is shown for both (A) water and (B) tadpole samples (*n* = 10 for each bar). The average amount of Bd detected per treatment in Bd-positive samples for (C) water and (D) tadpoles is shown with error bars (±SE) for treatments with three or more positive samples.

*Daphnia magna* density affected both the amount of Bd detected in the water (*X*^2^ = 7.15, *P* = 0.008; Table 1, Fig. 2C) and the proportion of tadpole samples positive for Bd (*X*^2^ = 6.53, *P* = 0.011; Table 1, Fig. 2B). Grazing period affected the proportion of water and tadpole samples positive for Bd and the amount of Bd detected in water samples (*P* < 0.001 for all comparisons; Table 1, Fig. 2). Likelihood ratio tests suggested that inclusion of the grazing period × *Daphnia* density interaction did not significantly improve model fit. Therefore, this term was removed from both models during model selection following the procedure in Zuur et al. (2009).

**Table 1:** Summary of statistical findings from the reduced zero-inflated negative binomial models

Experiment	Sample type	Infection measurement	Predictor	Test statistic	df	*P*
1: *Daphnia magna* at five densities grazing 5 or 72 h^1^	Water	Proportion	*Daphnia* density	*X*^2^ = 2.32	1	0.128
			Grazing period	*X*^2^ = 77.06	1	<0.001
		Amount	*Daphnia* density	*X*^2^ = 7.15	1	0.008
			Grazing period	*X*^2^ = 43.45	1	<0.001
	Tadpole	Proportion	*Daphnia* density	*X*^2^ = 6.53	1	0.011
			Grazing period	*X*^2^ = 12.01	1	0.001
		Amount	Grazing period	*X*^2^ = 2.50	1	0.114
2: *Daphnia dentifera* at five densities grazing 5 or 72 h^1^	Water	Proportion	*Daphnia* density	*X*^2^ = 13.76	1	<0.001
			Grazing period	*X*^2^ = 64.16	1	<0.001
		Amount	Grazing period	*X*^2^ = 50.82	1	<0.001
	Tadpole	Proportion	Grazing period	*X*^2^ = 69.46	1	<0.001
		Amount	*Daphnia* density	*X*^2^ = 3.00	1	0.083
			Grazing period	*X*^2^ = 6.29	1	0.012
3: Presence/absence of *D. dentifera* varying food density^2^	Water	Amount	*Daphnia* density	*X*^2^ = 8.17	1	0.004
			Food density	*X*^2^ = 4.53	1	0.033
			*Daphnia* density x Food density	*X*^2^ = 6.21	1	0.013
	Tadpole	Proportion	*Daphnia* density	*X*^2^ = 7.70	1	0.005
		Amount^3^	*Daphnia* density	*X*^2^ = 10.80	1	0.001
			*Daphnia* density x Food density	*X*^2^ = 9.28	1	0.002

1 Initial models for experiments 1 and 2 included *D. magna* or *D. dentifera* density (respectively), grazing period, and the interaction term.

2 Initial model for experiment 3 included food level, *D. dentifera* density, and the interaction term.

3 The results for amount of Bd detected in tadpole samples in experiment 3 were driven by a single sample. When we removed this sample, there were no significant predictors for this response.

Across species (comparing 0 *Daphnia*, 25 *D. magna*, and 25 *D. dentifera*), we found that treatments with 25 *D. magna* or 25 *D. dentifera* both reduce the amount of Bd detected in water samples (*F*_2,26_ = 4.39, *P* = 0.023; Table 2, Fig. 2C). A post hoc test showed no difference between 25 *D. magna* and 25 *D. dentifera* in terms of the amount of Bd detected in water (*P* = 086; Fig. 2C). Treatments differed significantly in the proportion of tadpoles that became infected (*X*^2^ = 11.62, *P* = 0.003, Table 2, Fig. 2B). A post hoc test revealed that *D. magna* reduced the proportion of positive tadpole samples compared with the control treatments, while *D. dentifera* did not (Bonferroni corrected α = 0.0167; comparing 0 *Daphnia* with 25 *D. magna*: *X*^2^ = 5.94, *P* = 0.015; comparing 0 *Daphnia* with 25 *D. dentifera*: *X*^2^ = 0.966, *P* = 0.326; Fig. 2B). The proportion of water samples that were positive for Bd and the amount of Bd detected in tadpoles did not differ among these three treatments (see Table 2).

**Table 2:** Comparisons between treatments in experiment 1 containing 0 *Daphnia*, 25 *D. magna*, or 25 *D. dentifera*

Sample type	Infection measurement	Test statistic	df	*P*
Water	Proportion	*X*^2^ = 2.27	2	0.322
	Amount	*F* = 4.39	2,26	0.023^1^
Tadpole	Proportion	*X*^2^ = 11.62	2	0.003^2^
	Amount	*F* = 0.09	2,12	0.914

1 A Tukey's HSD test revealed no difference between the treatments with 25 *D. magna* and those with 25 *D. dentifera*. However, both treatments with *Daphnia* had less Bd than the 0 *Daphnia* treatment.

2 Post hoc tests revealed that treatments with 25 *D. dentifera* did not differ from the 0 *Daphnia* treatment. However, treatments with 25 *D. magna* had a smaller proportion of samples testing positive for Bd compared with the 0 *Daphnia* treatment and the treatment with 25 *D. dentifera*.

### Experiment 2

**Figure 3:**
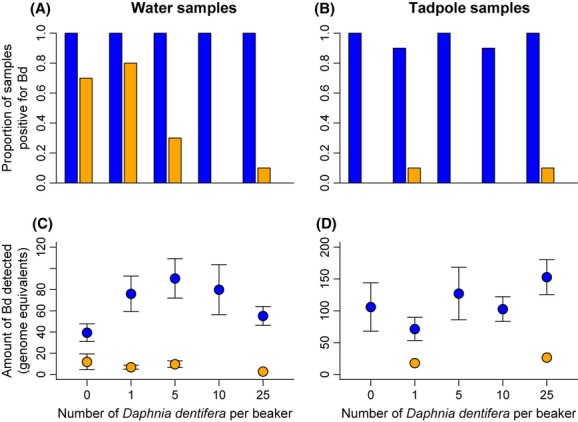
Results from experiment 2. Infection prevalence and amount of *Batrachochytrium dendrobatidis* (Bd) detected in samples from experiment 2 where we varied the number of *Daphnia dentifera* per beaker. Proportion of samples positive for Bd is shown for both (A) water and (B) tadpole samples (*n* = 10 for each bar). The average amount of Bd detected per treatment in Bd-positive samples for (C) water and (D) tadpoles is shown with error bars (±SE) for treatments with three or more positive samples. Dark (blue) bars and points represent the 5-h grazing period while light (orange) bars and points represent the 72-h grazing period. After the grazing period, we exposed all tadpoles to grazed water for 24 h.

*Daphnia dentifera* density influenced the proportion of water samples positive for Bd (*X*^2^ = 13.76, *P* < 0.001; Table 1, Fig. 3A), but did not significantly influence the proportion of tadpoles positive for Bd, or the amount of Bd detected in water or tadpoles (see Table 1, Fig. 3B−D). The proportion of samples positive for Bd and the amount of Bd detected in samples were always lower in the 72-h grazing period compared with the 5-h grazing period (*P* < 0.02 for all comparisons, Fig. 3). Based on likelihood ratio tests (see Methods), we removed the grazing period x *Daphnia* density interaction term from both models during model selection. Figure S1 shows a comparison between the common treatments from experiments 1 and 2.

### Experiment 3

**Figure 4:**
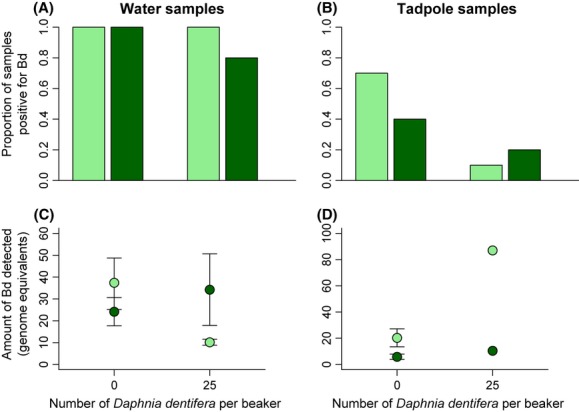
Results from experiment 3. Infection prevalence and amount of *Batrachochytrium dendrobatidis* (Bd) detected in samples from experiment 3. Dark bars and points represent the high-food treatments while light bars and points represent the low food treatments. Grazing period was 24 h for all treatments. Proportion of samples positive for Bd is shown for both (A) water and (B) tadpole samples (*n* = 10 for each bar). The average amount of Bd detected per treatment in Bd-positive samples for (C) water and (D) tadpoles is shown with error bars (±SE) for treatments with three or more positive samples.

The proportion of water samples positive for Bd was not significantly affected by *Daphnia* or algae treatment (Fig. 4A). However, the amount of Bd detected in water samples was affected by the interaction between *D. dentifera* density and food density (*X*^2^ = 6.21, *P* = 0.01; Table 1, Fig. 4C). When no *D. dentifera* were present, there was less Bd in water when concentrations of algae were higher, but when *D. dentifera* were present there was less Bd detected in water when densities of algae were lower. The proportion of tadpole samples positive for Bd was lower when more *D. dentifera* were present (*X*^2^ = 7.70, *P* = 0.005), but was not affected by food density ( see Table 1, Fig. 4B). The amount of Bd detected in tadpole samples was affected by the interaction between *D. dentifera* and food density (*X*^2^ = 9.28, *P* = 0.002; Table 1, Fig. 4D). However, this significant interaction was driven by a single sample in the 25 *Daphnia*, low-food treatment. When we removed this point, neither *Daphnia* density nor the interaction was significant predictors of the amount of Bd detected in tadpoles.

## Discussion

Our results demonstrate that direct predation upon parasites can reduce density of parasites in the environment and infection in hosts. Specifically, we found that *Daphnia* can reduce Bd levels in water and infection in tadpoles, but this effect was context-dependent. *Daphnia* abundance, *Daphnia* species identity, food concentration, and grazing period all affected the ability of *Daphnia* to reduce Bd in water and tadpole samples. Therefore, caution is warranted in assuming that *Daphnia* can successfully reduce infection in amphibians in natural systems.

In experiment 1, we found that high densities of both *Daphnia* species reduced Bd in water samples (see Fig. 2C, 3A), as previously demonstrated by Hamilton et al. (2012) and Woodhams et al. (2011). We also show that *Daphnia* can reduce tadpole infection (see Fig. 2B), but this effect only occurred for one of the two species we used in this study; direct comparison between the two *Daphnia* species (see Table 2) showed that only *D. magna* were able to reduce infection in tadpoles. As both species reduced the amount of Bd detected in water samples, this indicates that these species have similar rates of Bd consumption. However, zoospores may survive gut passage but be damaged and unable to infect tadpole hosts. *Daphnia magna* are larger than *D. dentifera,* and therefore are able to filter more water in a given time period (Burns 1969). Thus, zoospores are more likely to be consumed multiple times by *D. magna* than by *D. dentifera*. Therefore, *D. magna* may reduce infectiousness of zoospores at a greater rate than *D. dentifera*, even if the relative rates of Bd digestion are similar. Alternatively, Bd zoospores do not have thick cell walls or sheaths (Longcore et al. 1999), which suggests that they should not be particularly digestion resistant. Therefore, other mechanisms may drive the different effects of these two *Daphnia* species. Our results suggest that, when studying the effects of *Daphnia* on Bd in the field, it is important to consider the species identity and size of the *Daphnia* that are present.

High densities of *Daphnia* were able to reduce Bd in water and tadpole samples, but not in all circumstances. While the *Daphnia* densities we used in this experiment are within the range of densities found in natural systems, our highest densities (25 *Daphnia* per beaker; 100 *Daphnia* per L) and the highest densities in previous studies (1400 *Daphnia* per L; Hamilton et al. 2012; 1600 *Daphnia* per L; Woodhams et al. 2011) were likely above most natural densities. Field densities of *Daphnia* can occasionally reach over 100 individuals per L (e.g., ∼150: Luecke et al. 1990; up to 104: DeMott and Gulati 1999), but many field surveys have reported maximum densities below 50 individuals per L (Kwik and Carter 1975; DeMott 1983; Dawes et al. 1987). Thus, densities as high as those found in our 25 *Daphnia* treatments and as those used in previous studies are unlikely to be commonly found in nature. Limitations on *Daphnia* abundance from competition or predation may decrease the likelihood of *Daphnia* reducing Bd infection in natural systems.

In the absence of *Daphnia*, we found that the amount of Bd detected in water was lower when concentrations of algae were higher (see Fig. 4C). This pattern suggests a direct negative interaction between algae and Bd zoospores. This could occur if high concentrations of algae interfere with the ability of zoospores to swim through water through physical interference. Alternatively, some green algae exhibit allelopathy (Wolfe and Rice 1979), so *A. falcatus* may release chemicals that directly kill or impair Bd. Future studies are necessary to understand the direct impacts of algae on Bd zoospores. We also found that when *Daphnia* were present, the amount of Bd detected in water samples showed a different pattern; in this case, Bd was higher when densities of algae were higher (see Fig. 4C). It is possible that this pattern is driven by food saturation in the high-food treatments, where *Daphnia* were unable to consume all the algae and Bd in the water. However, individual *D. dentifera* under these conditions can filter over 10 mL water per day (Hall et al. 2010), so it is likely that the entire contents of our beakers would have been filtered at least once during the 24-h experiment. Gut passage time and food assimilation in *Daphnia* vary with food density; when food densities are low, gut passage time increases and assimilation efficiency of field-collected algae increases (DeMott et al. 2010). Therefore, even if *Daphnia* in the low-food treatments consumed the same number of zoospores as in the high-food treatments, a greater proportion of those zoospores may have been digested. This indicates that zoospores may be better able to survive passage through the gut of a *Daphnia* in high-food conditions. Alternatively, *Daphnia* can exhibit selective grazing (Burns 1968; Porter 1973; Haney 1987), so high densities of algae could have led to *Daphnia* consuming fewer Bd zoospores if *A. falcatus* is their preferred food. These results have implications for Bd disease risk in natural systems. In eutrophic lakes, for example, high densities of algae may have direct negative effects on Bd zoospores, reducing disease risk for amphibians. However, high densities of algae may reduce digestion of Bd zoospores, which would create an indirect positive effect of algae on Bd. It is unknown how these conflicting forces will affect Bd levels in eutrophic environments.

In both experiments 1 and 2, we found that treatments with longer grazing periods almost always reduced Bd in our samples. As we saw this pattern across all *Daphnia* densities (including treatments with no *Daphnia*), this is unlikely due to effects of *Daphnia* grazing. Bd zoospores were only added once at the beginning of the grazing period and have a limited lifespan in water (Piotrowski et al. 2004). Therefore, it is likely that we detected less Bd in water samples after 72 h due to increased time for zoospore mortality compared with the 5-h grazing periods. When zoospores die, their cells and DNA degrade, resulting in lower qPCR values. Additionally, when tadpoles were added after the beakers after 72 h of grazing, there were fewer surviving zoospores able to infect the tadpoles. It is possible that we would have found different results had we exposed tadpoles to Bd while simultaneously allowing *Daphnia* to graze. The ability of *Daphnia* to consume Bd in natural systems is likely affected by the length of time that zoospores remain in the water before finding hosts. Thus, if zoospores are able to find hosts quickly, then the effects of *Daphnia* grazing may be limited.

Another notable trend we observed is that patterns found in water samples were not necessarily similar to the observed patterns in tadpoles from the same experiment. For example, in both experiments 1 and 2, *D. dentifera* reduced Bd in water samples, but had no effect on tadpole samples. This indicates that infection in tadpoles is not necessarily dose-dependent. A previous experimental study demonstrated that only one of three amphibian species tested exhibited a dose-dependent response to Bd (Gervasi et al. 2013). Multiple factors may be involved in determining infection in tadpoles. For example, there is variation within amphibian species in anti-Bd microbial defenses (Harris et al. 2006; Lam et al. 2010), and different species exhibit behaviors that affect their chances of becoming infected (Rowley and Alford 2007). These factors may have large effects on Bd infection in tadpoles and sometimes outweigh the effects of zoospore densities. Therefore, it is essential to monitor Bd in both water bodies and amphibian hosts.

Our study demonstrated that direct predation on parasites can reduce infection of a deadly fungal parasite responsible for amphibian population declines and extirpations around the globe. However, this effect was context-dependent and varied with predator species, predator density and resource availability. Therefore, it cannot be assumed that predators will successfully act as biocontrol agents for infectious diseases, even if they have the ability to consume parasites. When attempting to understand the effects predation upon parasites, numerous biotic and abiotic conditions must be considered.

## Supporting Information

**Figure S1.** Infection prevalence and amount of Bd detected in common treatments from experiments 1 and 2. Dark (blue) bars and points represent the 5 h grazing period and light (orange) bars and points represent the 72 h grazing period. Amount of Bd detected is shown as the average level for Bd-positive samples per treatment (±SE). Points without error bars were treatments with fewer than three positive samples.

**Figure S2.** Average whole-body wet mass of tadpoles used in each experiment (±SE). The number of tadpoles used in each experiment varied (experiment 1: *n* = 120, experiment 2: *n* = 100, experiment 3: *n* = 40).

**Data S1.** Methods and results.
