# Supplementary material for: Daphnia predation on the amphibian chytrid fungus and its impacts on disease risk in tadpoles
Source: Ecol Evol. 2013 Sep 23;3(12):4129–38. doi: 10.1002/ece3.777 (PMC3853558; doi:10.1002/ece3.777)
Supplement: Supplementary file 1 [file ece30003-4129-SD1.docx]

**Figure S1**


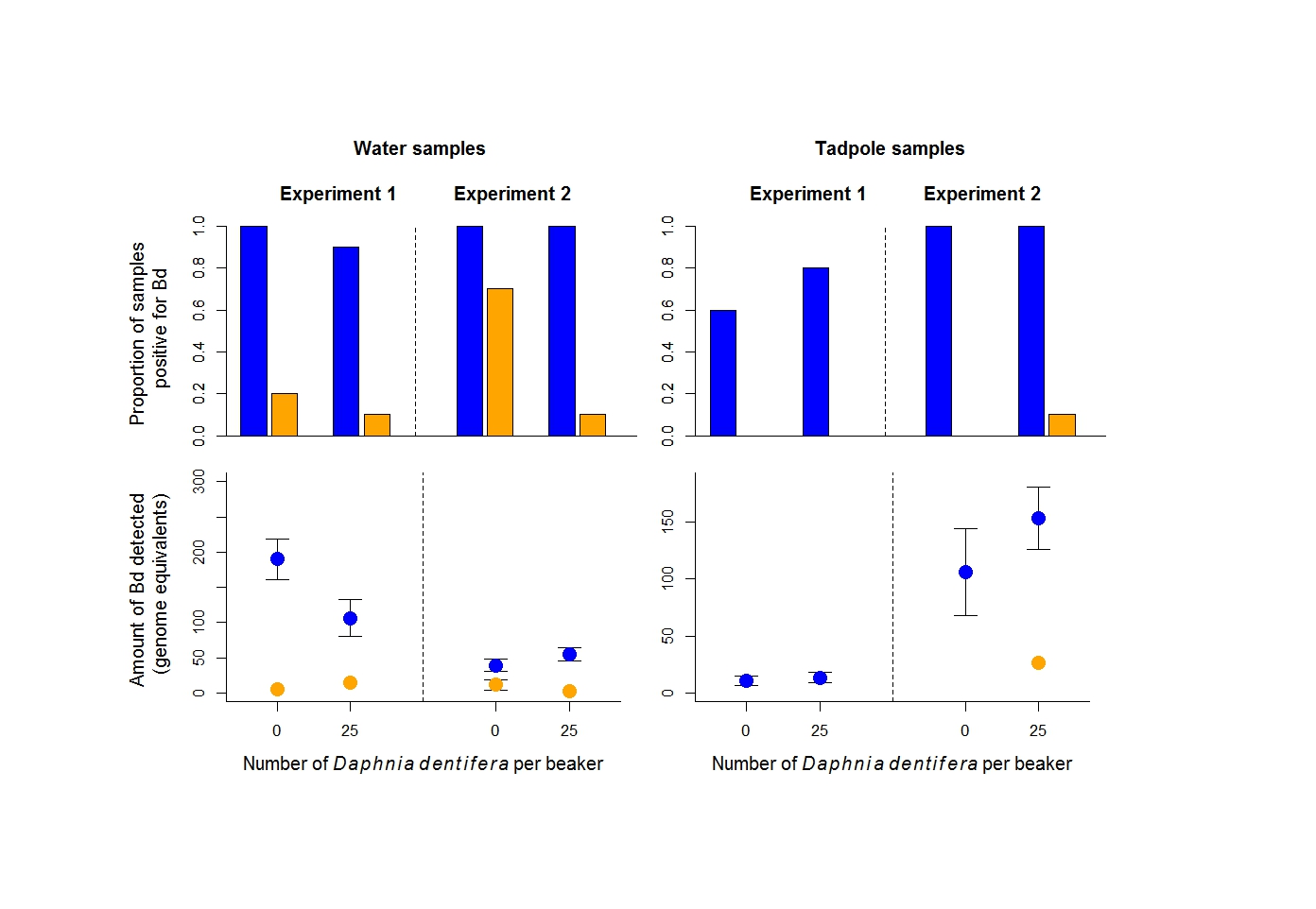


Figure S1: Infection prevalence and amount of Bd detected in common treatments from experiments 1 and 2. Dark (blue) bars and points represent the 5 hour grazing period and light (orang) bars and points represent the 72 hour grazing period. Amount of Bd detected is shown as the average level for Bd-positive samples per treatment (±SE). Points without error bars were treatments with fewer than three positive samples.

**Supplemental methods and results**

Because experiments 1-3 were not conducted concurrently, we compared the mass of tadpoles among experiments using an ANOVA. Mass of tadpoles differed among experiments (F_2,257_=13.69, p <0.001; Figure S2). A Tukey’s test revealed that tadpoles from experiments 1 and 2 did not differ in size, but tadpoles in experiment 3 were smaller than those in the first two experiments.

**Figure S2**

**
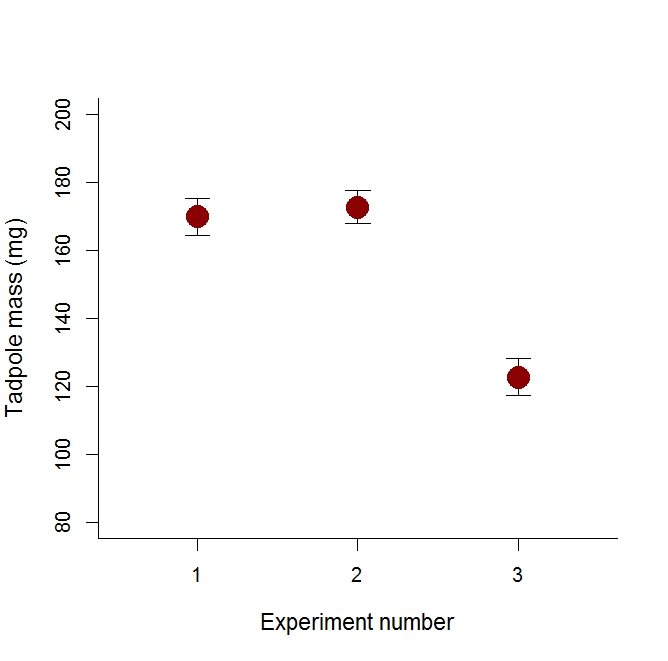
**

Figure S2: Average whole-body wet mass of tadpoles used in each experiment (±SE). The number of tadpoles used in each experiment varied (experiment 1: n = 120, experiment 2: n = 100, experiment 3:n = 40).
